# How can nanomicelle-curcumin modulate aluminum phosphide-induced neurotoxicity?: Role of SIRT1/FOXO3 signaling pathway

**DOI:** 10.3934/Neuroscience.2023005

**Published:** 2023-04-04

**Authors:** Milad Khodavysi, Nejat Kheiripour, Hassan Ghasemi, Sara Soleimani-Asl, Ali Fathi Jouzdani, Mohammadmahdi Sabahi, Zahra Ganji, Zahra Azizi, Akram Ranjbar

**Affiliations:** 1 Department of Pharmacology and Toxicology, School of Pharmacy, Medicinal Plants and Natural Products Research Center, Hamadan University of Medical Sciences, Hamadan, Iran; 2 Research Center for Biochemistry and Nutrition in Metabolic Diseases, Kashan University of Medical Sciences, Kashan, Iran; 3 Department of Clinical Biochemistry, Abadan University of Medical Sciences, Abadan, Iran; 4 Department of Anatomical Sciences, Faculty of Medicine, Hamadan University of Medical Sciences, Hamadan, Iran; 5 Neuroscience and Artificial Intelligence Research Group (NAIRG), Student Research Committee, Hamadan University of Medical Sciences, Hamadan, Iran; 6 USERN office, Hamadan University of Medical Sciences, Hamadan, Iran; 7 Department of Neurosurgery, University of Pittsburgh Medical Center, Pittsburgh, PA, USA

**Keywords:** aluminum phosphide, brain, curcumin, nanomicelle-curcumin, oxidative stress

## Abstract

Aluminum phosphide (ALP) is among the most significant causes of brain toxicity and death in many countries. Curcumin (CUR), a major turmeric component, is a potent protective agent against many diseases, including brain toxicity. This study aimed to examine the probable protection potential of nanomicelle curcumin (nanomicelle-CUR) and its underlying mechanism in a rat model of ALP-induced brain toxicity. A total of 36 Wistar rats were randomly divided into six groups (n = 6) and exposed to ALP (2 mg/kg/day, orally) + CUR or nanomicelle-CUR (100 mg/kg/day, orally) for 7 days. Then, they were anesthetized, and brain tissue samples were dissected to evaluate histopathological alterations, oxidative stress biomarkers, gene expression of SIRT1, FOXO1a, FOXO3a, CAT and GPX in brain tissue via hematoxylin and eosin (H&E) staining, biochemical and enzyme-linked immunosorbent assay (ELISA) methods and Real-Time PCR analysis. CUR and nanomicelle-CUR caused significant improvement in ALP-induced brain damage by reducing the MDA levels and induction of antioxidant capacity (TTG, TAC and SOD levels) and antioxidant enzymes (CAT, GPX), modulation of histopathological changes and up-regulation of gene expression of SIRT1 in brain tissue. It was concluded that nanomicelle-CUR treatment ameliorated the harmful effects of ALP-induced brain toxicity by reducing oxidative stress. Therefore, it could be considered a suitable therapeutic choice for ALP poisoning.

## 1. Introduction

Aluminum phosphide (ALP) is a highly toxic inorganic compound used as a rodenticide, insecticide and fumigant for stored cereal grains. ALP is among the most significant causes of suicidal poisoning in many countries [1],[2]. ALP tablets release phosphine gas (PH3) after moisture exposure, and it is rapidly absorbed by inhalation, skin or intestinal tract. Acute toxicity caused by ALP exposure is detected in the heart, lungs, liver, brain, kidney and central nervous system [3],[4]. However, the exact toxicity mechanism of phosphine gas remains unknown. As a highly reactive radical, phosphine is released after hydrolysis of ALP and diffused to the intracellular compartment, causing cellular damage by oxidative damage. [5]. Oxidative stress has been reported to be the most crucial ALP-induced cytotoxicity. The toxicity mechanism of phosphine involves cytochrome C oxidase inhibition in the mitochondria and the formation of highly reactive hydroxyl radicals leading to cellular hypoxia [6]. According to the microscopic investigation, ALP impacts the cerebral and cerebellar cortex of the human brain in distinct ways. ALP caused the cortical layers to become disorganized; round-shaped neurons have concave borders; Nissl granules have degraded; an eccentric nucleus has deeply stained and degenerated [7]. ALP Exposure decreased the catalase (CAT) and glutathione peroxidase (GPX) activity [8],[9]. It also increased the superoxide dismutase (SOD), hydrogen peroxide (H2O2), reactive oxygen species (ROS), and malondialdehyde (MDA) levels [1],[2],[10]. Curcumin (CUR), a yellow pigment from Curcuma longa, is a major component of turmeric and is commonly used as a spice and food coloring. CUR is a low molecular weight polyphenol with the molecular formula C21H20O6 [11]. Therapeutic properties of CUR are considered to be anti-oxidant, anti-inﬂammatory, proapoptotic, chemopreventive, anti-proliferative, and anti-tumor properties [12].

Several studies have reported that CUR inhibits a variety of reactive oxygen species (ROS) such as superoxide anion, hydroxyl radicals and antioxidant activities of SOD, GSH, glutathione-s-transferase (GST) and glutathione peroxidase (GPx) in a variety of cancers [13]. It has been shown that CUR crosses the blood-brain barrier and has high antioxidant effects on the brain. Curcumin protects DNA and proteins from oxidative damage in atherosclerosis and neurological disorders by preventing lipid peroxidation [14],[15]. CUR displays regulatory effects on the activity of Na^+^, K^+^-ATPase in brain microsomes [16]. It interacts with human serum albumin (HSA), the essential transport protein in the human plasma [17]–[19]. However, the benefits of CUR have been impaired by its low water solubility, low absorption, rapid metabolism and clearance in the body [20]. CUR endures quick hydrolytic degeneration at pH values above neutral and is empirically insoluble in neutral or lower pH water [21]. This low water solubility restricts extra pharmacological promotion and feasible administration of the curcumin [22]. So, more curcumin is needed to provide its expected effect [23]. Developing nanocarriers that increase curcumin's water solubility and distribution in tissues is proposed to resolve this issue [24]. According to studies, nano-curcumin can improve the antioxidant, anti-inflammation, antiproliferative and neuroprotective properties of curcumin [25]. The nanocarriers comprise liposomes, micelles, magnetic nanoparticles, dendrimers and polymeric or lipid-based carriers [26]. In addition, nanoparticle micelles are efficient tools for drug encapsulation to increase water solubility and biological availability. Clinically, these prevent rapid uptake and clearance by mononuclear phagocytes [27]. Given the lack of studies exploring curcumin's mechanisms of action, we investigated the effect of curcumin and nanomicelle-CUR on ALP-induced brain damage in rats with oxidative stress biomarkers, gene expression of antioxidant enzymes, histological changes and mRNA expression levels within the silent information regulator 1 (SIRT1)/FOXO3 signaling cascade in ALP treated brain tissue.

## 2. Materials and methods

### 2.1. Reagents

ALP > 95% purity was prepared from Samiran Pesticide Formulating Co. (Tehran, Iran). Native CUR and nanomicelle-CUR (CUR C3 complex-loaded nanoparticles) were bought from Exir Nano Sina Co., Tehran, Iran (IRC: 1228225765). There is 100% encapsulation of curcumin in nanomicelles, which are about 10 nm. ALP, CUR and nanomicelle-CUR were stored at 4 °C. The other chemicals for biochemistry were purchased from Sigma-Aldrich Co. (St. Louis, Missouri, USA). The chemicals used for the experiments had an analytical grade.

### 2.2. Experimental animals

Thirty-six Wistar male rats with an average weight of 235 ± 15 g were obtained from the Animals Lab of Hamadan University of Medical Sciences. The rats were kept in standard plastic laboratory cages at room temperature (25 ± 2 °C) and humidity (60 ± 5%) with 12 hours light/dark cycle. During the experimental study, the rats were fed with standard chow and tapped water ad libitum. After one week of adaptation to the laboratory conditions, the rats were divided randomly into six groups, including control and five treatment groups. The five treatment groups were treated with CUR (100 mg/kg/day), nanomicelle-CUR (100 mg/kg/day), ALP (2 mg/kg/day), ALP (2 mg/kg/day) + CUR (100 mg/kg/day) and ALP (2 mg/kg/day) + nanomicelle -CUR (100 mg/kg/day). All materials were given orally by gavage for seven days. All procedures were approved in advance by the Ethical Committee for Hamadan University of Medical Sciences (IR.UMSHA.REC: 9704262220).

### 2.3. Preparation of samples

The animals were euthanized under deep anesthesia with ketamine (50 mg/kg) 24 hours after the last treatment. All the animals were sacrificed separately in one day. After complete anesthesia with ketamine/xylazine, brain tissues were immediately removed, dissected over ice-cold glass and stored at −80 °C until tests. A saline solution was used to clean the brain tissues as soon as they were separated. In addition to histopathological studies, one hemisphere was frozen in liquid nitrogen and stored at −70 °C for biochemical analyses. 100 mg of homogenized tissues were mixed with 1 ml of lysis buffer (HEPES [10 mM], KCl [10 mM], MgCl2 [1.5 mM], EDTA [1 mM], Triton X-100 (0.2%) and DTT [0.5 mM]) in 7.9 pH) and incubated in ice for 20 min. As part of the homogenization process, brain allocations were homogenized in 1:1 volumes of PBS (pH 7.4). A Teflon glass homogenizer was used to homogenize tissues. Afterwards, the homogenate was centrifuged at 10,000 g for 30 minutes at 4 °C. The supernatant was stored at −80 °C as brain homogenate for additional biochemical assays.

### 2.4. Assessment of biochemical parameters

#### 2.4.1. Measurement of total protein

We measured total protein concentration using the Bradford reagent. The Bradford reagent was prepared by dissolving 100 mg of Coomassie brilliant blue G-250 in 50 mL of 95% ethanol and adding 100 mL of 85% phosphoric acid to the solution. To measure the protein content of the homogenates, we used bovine serum albumin at 595 nm [28].

#### 2.4.2. Measurement of lipid peroxidation (LPO)

LPO was performed by the Yagi method using spectrophotometry. In this method, 100 µl of the sample was mixed with 100 µl of SDS, 1.5 ml of acetic acid, 1.5 ml of TBA and 200 µl of water. The reaction mixture was placed in a 95 °C bath for 60 min. After water cooling, 3 ml of butanol was added and centrifuged at 1000 g, and the butanol layer was removed. The MDA concentration was measured using a UV-Visible Spectrophotometer at 532 nm [29].

#### 2.4.3. Measurement of total thiol groups (TTG)

TTG were determined by the method of Rao et al. [30]. To measure total thiol, 50 µl of the sample was mixed with 150 µl of Tris-EDTA buffer (0.302 g Tris base, up to 8 ml of EDTA, pH 10.2), 10 µl of DTNB and 790 µl of methanol and then incubated for 15 min at room temperature and centrifuged at 1000 g for 10 min. The supernatant absorbance was measured at 412 nm using a UV-Visible Spectrophotometer.

#### 2.4.4. Measurement of total antioxidant capacity (TAC)

TAC was measured using the method of Benzie [31]. The FRAP (ferric reducing ability of plasma) method measured total antioxidant capacity. It is performed based on the antioxidant power of brain tissue to deoxidize Fe3+ to Fe2+. Briefly, 100 µl of homogenized tissue supernatant tissue was added to 3 ml of FRAP reagent. In FRAP reagent, 25 ml acetate buffer (300 mM, pH 3.6) is mixed with 16 ml acetic acid per buffer unit, 2.5 ml TPTZ solution (TPTZ 10 mM in HCl 40 mM) and 2.5 ml FeCl3.6H2O. After vortexing and incubating for 10 minutes at 37 °C, the resulting solution's absorption at 593 nm was measured. Optical density was measured according to a calibration curve attained by serial concentrations of FeSO4 at 593 nm.

### 2.5. Molecular parameters

#### 2.5.1. Determination of mRNA expression of SIRT1, FOXO1, FOXO3, CAT and GPX in the brain tissue

Total RNA extraction was performed manually from tissues by RNX-Plus reagent (Cinnagen, Tehran, Iran). Complementary DNA (cDNA) synthesis was carried out by the PrimeScript RT reagent kit (TaKaRa Biotechnology, Japan). Quantitative Real-Time PCR was performed with SYBR premix Ex Taq™ ^II^ (TaKaRa Biotechnology, Japan) on a Roche LightCycler 96 System (Roche Life Science Deutschland GmbH, Sandhofer, Germany). The characteristics of forward and reverse primer sequence (5′ →3′) were as follows. β-Actin: forward CCCGCGAGTACAACCTTCT and reverse CGTCATCCATGGCGAACT. SIRT1: forward CAGTGTCATGGTTCCTTTGC and reverse CACCGAGGAACTACCTGAT. FOXO1a: forward CGAGTGGATGGTGAAGAGTG and reverse CGAATAAACTTGCTGTGTAGGG. FOXO3a: forward CTCCCGTCAGCCAGTCTATG and reverse GCTTAGCACCAGTGAAGTTCC. CAT: forward CCCAGAAGCCTAAGAATGCAA and reverse TCCCTTGGCAGCTATGTGAGA. GPX: forward CACTGTGGCTGAGCTGTTGT and reverse CCAAGCAATTCAAGCCTCT. The 2 -ΔΔCT formula was used [32] to investigate the fold change in gene expression. Then, 10 µl of H2O diluted sample was added to 300 ml reagent warmed at 37 °C. Then, 100 µL of brain tissue sample was added to 3 ml of FRAP reagent. The complex between TPTZ and Fe2+ makes a blue color with 593 nm absorbance.

#### 2.5.2. Superoxide dismutase (SOD) assessment

SOD enzyme activity assay was based on inhibiting nitroblue tetrazolium (NBT) reduction due to O_2_^−^ generation by the xanthine/xanthine oxidase system. One unit of SOD activity was defined as the amount of SOD enzyme causing 50% inhibition of the NBT reduction rate. A competitive ELISA kit (SOD activity KiaZist, Iran) determined SOD activity by the manufacturer's instructions. The product was measured spectrophotometrically at 560 nm.

#### 2.5.3. Histopathologic examination

To observe brain tissue under a light microscope, brain samples were fixed with 10% formalin. The brain samples were embedded in paraffin after they had been tissue processed. Hematoxylin-eosin staining was applied to paraffin-embedded specimens with 5-mm-thick sections (H&E). Sections were examined under a Leica DFC280 light microscope under a Leica Q (DMLB 2/11888110) Win and Image Analysis System (Leica Micros Imaging Solutions Ltd, Cambridge, U.K.). An experienced histologist who was blind to the study groups and treatments carried out the microscopic assessments.

### 2.6. Statistical analysis

The SPSS 16.0 (SPSS Inc., Chicago, IL, USA) statistical program was used for the statistical analysis. Based on the results, the mean and standard deviation were calculated. Tukey's post hoc test was used to determine whether the data were significant after a one-way analysis of variance (ANOVA). P-value < 0.05 was regarded as a significant change in all the experiments.

## 3. Results

### 3.1. Comparison of the MDA levels between the study groups

**Figure 1.**
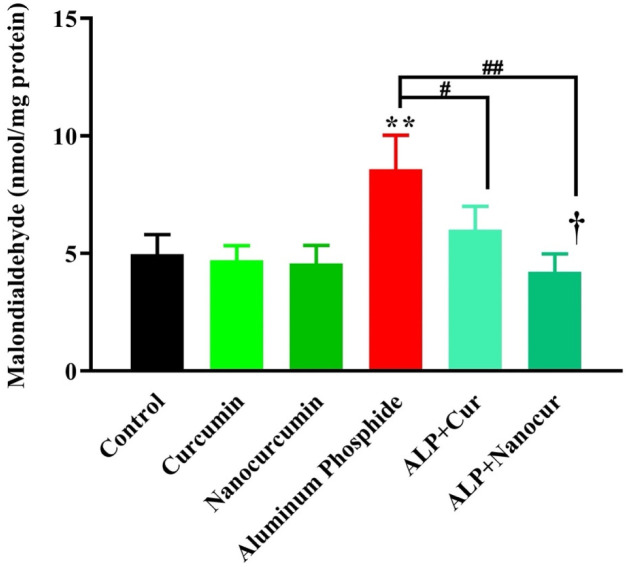
Comparison of the brain MDA levels between groups of male rats.

MDA levels differed significantly between the groups ((F (5, 30) = 17.56), P < 0.0001). According to Figure 1, the ALP group's MDA level of brain tissue was significantly increased compared to the control group (P < 0.01). The MDA levels were significantly decreased in the group's ALP + CUR groups (P < 0.05) and ALP + nanomicelle-CUR (P < 0.01) compared to the ALP group. The brain MDA levels in ALP + CUR and ALP + nanomicelle-CUR groups were not significantly different from the control group (P < 0.05). In addition, the CUR and nanomicelle-CUR groups had no significant difference compared with the control group (P > 0.05).

Results are mean ± SD, n = 6; ALP: Aluminum phosphate; N = 6. Cur: Curcumin. Nanocurcumin/NanoCur: Nanomicelle-curcumin. ANOVA and Tukey's post hoc test were used to compare brain MDA levels between the groups. The ALP group's MDA level increased compared with the control group (**P < 0.01). The MDA level was decreased in the ALP-CUR group compared with the ALP group (^#^P < 0.05). Also, the MDA level was reduced in the ALP-Nanomicelle-CUR group compared with the ALP group (^##^P < 0.01). In addition, the MDA level was decreased in the ALP + Nanomicelle-CUR group compared with the ALP + CUR group (^†^P < 0.05).

### 3.2. Comparison of the TTG levels between the study groups

**Figure 2.**
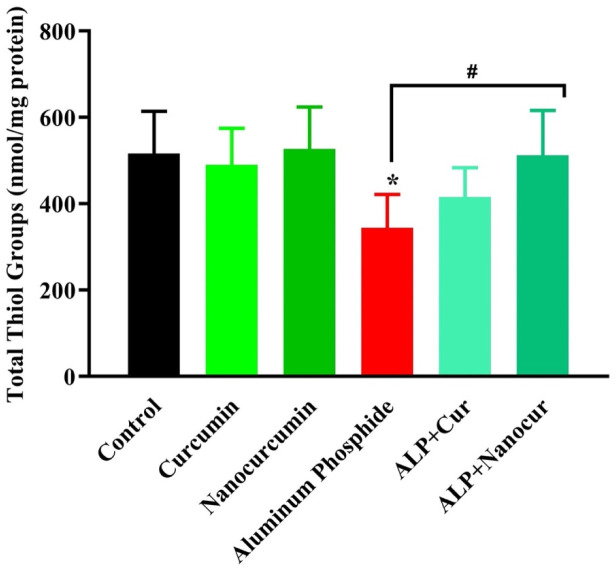
Comparison of the brain TTG levels between the male rat groups.

The TTG levels between the groups were significantly different (F (5, 30) = 3.988), P = 0.0068). According to Figure 2, the TTG level of brain tissue was significantly decreased in the ALP group compared to the control group (P < 0.05). In the ALP + nanomicelle-CUR group, the brain TTG level significantly increased compared to the ALP group (P < 0.05). In the ALP + CUR group, the tissue TTG level was not significantly different compared to the ALP group (P < 0.05). Also, ALP + CUR and ALP + nanomicelle-CUR groups did not show significant differences in the tissue TTG level compared to the control group (P > 0.05). In addition, there were no significant changes in the CUR and nanomicelle-CUR compared with the control group (P > 0.05).

Results are mean ± SD, N = 6; ALP: Aluminum phosphate. Cur: Curcumin. Nanocurcumin/NanoCur: Nanomicelle-curcumin. ANOVA and Tukey's post hoc test were used to compare the brain TTG levels between the groups. The brain TTG level was decreased in the ALP group compared with the control group (*P < 0.05). In addition, The TTG level was increased in the ALP+ Nanomicelle-CUR group compared with the ALP group (#P < 0.05).

### 3.3. Comparison of the TAC levels between the study groups

**Figure 3.**
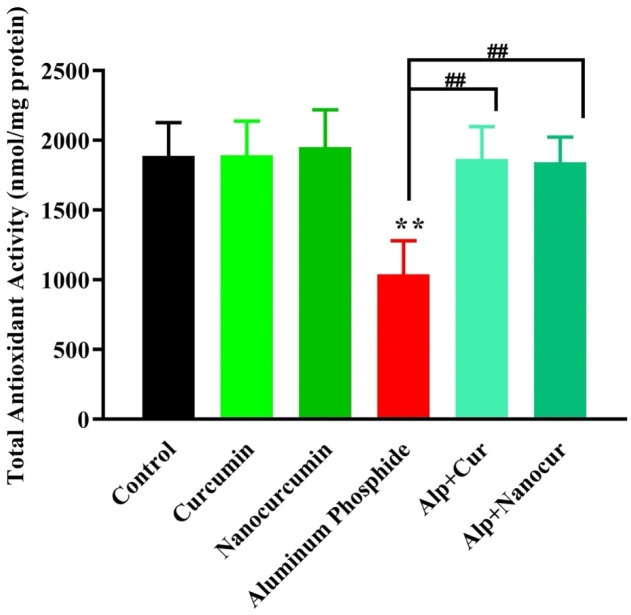
Comparison of the brain TAC levels between male rat groups.

There was a significant difference in TAC level between the groups (F (5, 30) = 13.01), P < 0.0001). As shown in Figure 3, the brain TAC level was significantly decreased in the ALP group compared to the control group (P < 0.01). The tissue TAC levels significantly increased in the ALP + CUR and ALP + nanomicelle-CUR compared to the ALP group (P < 0.01). Also, the TAC levels in the ALP + CUR and ALP + nanomicelle-CUR had no significant difference compared to the control group (P > 0.05). In addition, the TAC level had no significant difference in the CUR and nanomicelle-CUR groups compared with the control group (P > 0.05).

Results are mean ± SD, N = 6; ALP: Aluminum phosphate. Cur: Curcumin. Nanocurcumin/NanoCur: Nanomicelle-curcumin. ANOVA and Tukey's post hoc test were used to compare the brain TAC levels between the groups. The TAC level was decreased in the ALP group compared with the control group (**P < 0.01). In addition, the brain TAC level was increased in the groups of ALP + CUR and ALP + Nano micelle-CUR compared with the ALP group (##P < 0.01).

### 3.4. Comparison of the SOD levels between the study groups

**Figure 4.**
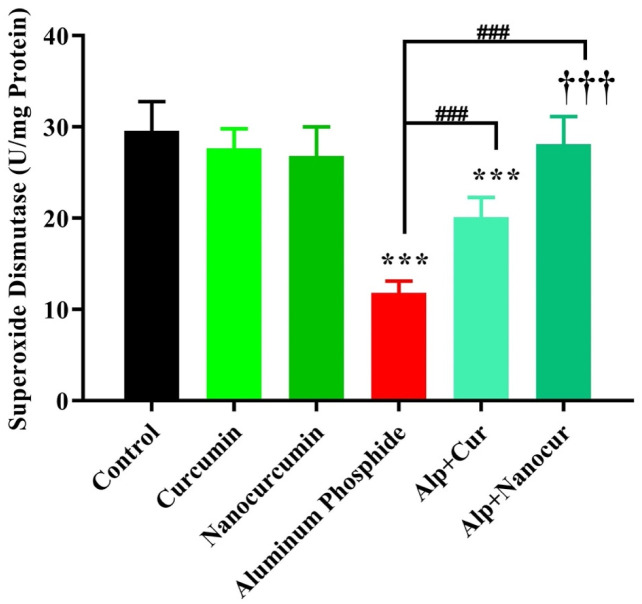
Comparison of the brain SOD levels between male rat groups.

There was a significant difference in SOD level between the groups (F (5, 30) = 41.25), P < 0.0001). Figure 4 showed that the brain SOD level was significantly decreased in the ALP group compared to the control group (P < 0.001). In the ALP + CUR groups and ALP + nanomicelle-CUR groups, the levels of tissue SOD were significantly increased compared with the ALP group (P < 0.001). There was no significant difference in tissue SOD level between the ALP + nanomicelle-CUR and control groups (P > 0.05). In contrast, its level in the ALP + CUR group was significantly decreased compared with the control group (P < 0.001). Also, the SOD level increased dramatically in the ALP + nanomicelle-CUR group compared with the ALP + CUR group (P < 0.001). In addition, the SOD levels had no significant difference in the CUR and nanomicelle-CUR groups compared with the control group (P > 0.05).

Results are mean ± SD, N = 6; ALP: Aluminum phosphate. Cur: Curcumin. Nanocurcumin/NanoCur: Nanomicelle-curcumin. ANOVA and Tukey's post hoc test were used to compare the brain SOD levels between the groups. The levels of SOD in the ALP and ALP + CUR groups decreased compared with the control group (***P < 0.001). Also, in the groups ALP + CUR and ALP + Nanomicelle-CUR, the SOD levels were increased compared with the ALP group (###P < 0.001). The levels of SOD were increased in the ALP + Nanomicelle-CUR group compared with the ALP + CUR group (†††P < 0.001).

### 3.5. The effects of curcumin and nanocurcumin on SIRT1, FOXO1a, FOXO3a, CAT and GPx gene expression in brain tissue

**Figure 5.**
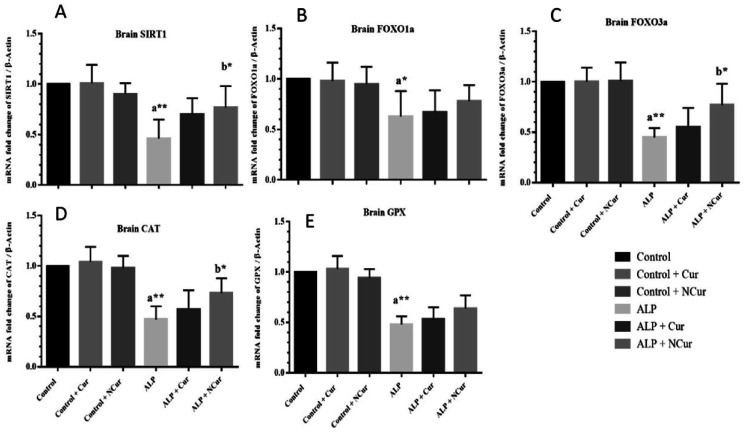
Comparison of the SIRT1, FOXO1a, FOXO3a, CAT and GPx gene expressions in brain tissue.

There was a significant difference in mRNA levels of SIRT1 (F (5, 30) = 13.7), P < 0.0001), FOXO1a (F (5, 30) = 5.184), P = 0.0015) and FOXO3a (F (5, 30) = 20.03), P < 0.0001) expression between the groups. As shown in Figure 5, the mRNA levels of SIRT1, FOXO1a and FOXO3a in the ALP group were significantly (P < 0.01, P < 0.05 and P < 0.01, respectively) lower than the control group. The SIRT1, FOXO1a and FOXO3a mRNA levels in the ALP + curcumin group were not significant differences from the ALP group. The mRNA level of SIRT1 and FOXO3a in the ALP + nanomicelle-CUR group had a significant (P < 0.05) increase compared to the ALP group. We did not find a significant difference among mRNA levels of FOXO1a in the ALP + nanomicelle-CUR group and in the ALP group. Also, there was a significant difference in levels of CAT (F (5, 30) = 13.19), P < 0.0001), GPx (F (5, 30) = 3.524, P = 0.0126) expression between the groups. The mRNA level of CAT in the ALP group was significantly (P < 0.001) lower than in the control group. This is while treatment with nanocurcumin significantly (P < 0.01) increased the expression of this gene compared to the ALP group. The mRNA expression of GPx was especially (P < 0.001) reduced in ALP-exposed rat brains. We did not find a significant difference among mRNA levels of GPx in ALP + nanomicelle-CUR, ALP + curcumin group with ALP group.

Results are mean ± SD, N = 6; ALP: Aluminum phosphate. Cur: Curcumin. NCur: Nanomicelle-curcumin. ANOVA and Tukey's post hoc test were used to compare the brain gene expression levels between the groups. Curcumin and nanomicelle Curcumin modulate SIRT1, FOXO1a and FOXO3a gene expression changes induced by ALP in brain tissue in rats. (A) SIRT1mRNA expression level. (B) FOXO1a mRNA expression level. (C) FOXO3a mRNA expression level. (D) CAT mRNA expression level. (E) GPx mRNA expression level. Results are mean ± SD, n = 8. The difference between the control and other groups is significant at P < 0.01 (aa) and P < 0.05 (a). The difference between ALP and other groups is significant at P < 0.05 (b).

### 3.6. Histological findings

**Figure 6.**
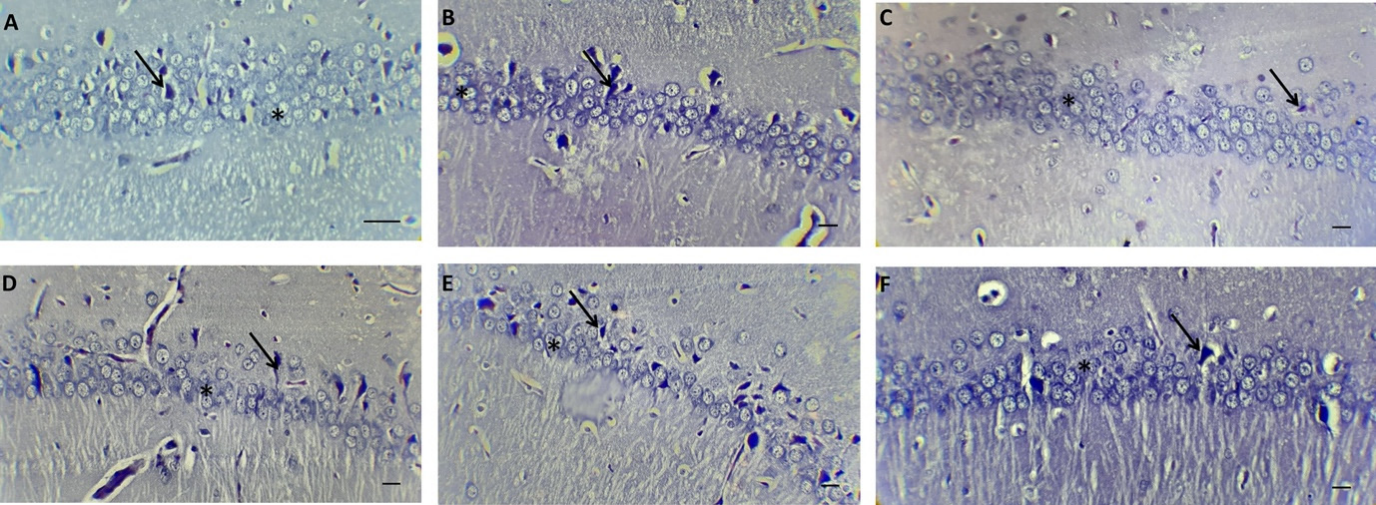
The morphology of the brain tissue. A: Control, B: CUR, C: Nanomicelle-CUR, D: ALP, E: ALP + CUR, F: ALP + Nanomicelle-CUR. The star shows healthy cells and flashes of pyknosis cells.

**Figure 7.**
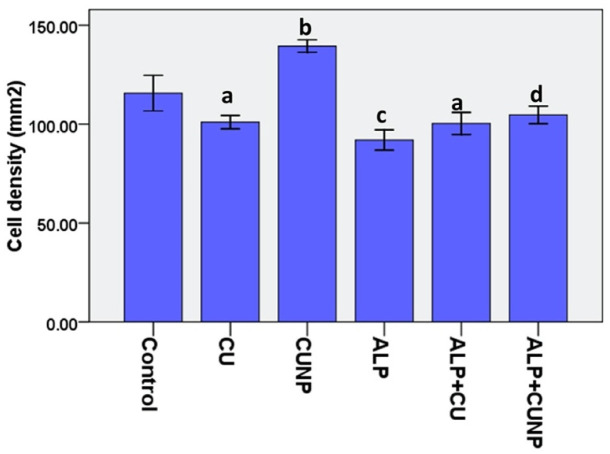
Histopathological examination of hippocampal CA1 area in groups.

The histopathological examinations of brain tissue from different groups can be seen in Figure 6. In a histological examination of the hippocampal CA1 area, healthy pyramidal cells are seen as euchromatin, but dead cells are pycnotic. There was a significant difference in the cell density between the groups (F (5, 30) = 239.4), P < 0.0001). Statistical analysis showed that the highest cell density belonged to the nanomicelle-CUR group, which was significant (p < 0.001) compared to all groups. Administration of CUR decreased the number of living cells compared to the control group (p < 0.01). Also, ALP administration significantly decreased cell density compared to the control group (p < 0.001). Administration of CUR with ALP did not affect cell number, while nanomicelle-CUR + ALP treatment significantly increased the number of living cells compared to the ALP group (p < 0.05) (Figure 7).

Results are mean ± SD, N = 3. The figure is representative of the nickel-stained sections of hippocampal CA1 from experimental groups. a: P < 0.01 compared to the control group, b: P < 0.001 compared to other groups, c: P < 0.001 compared to Nanomicelle-CUR and control groups, d: P < 0.05 compared to the ALP group.

## 4. Discussion

ALP exposure affects several neurobehavioral changes, including ataxia, stupor, tremors, nausea and vomiting, which may lead to convulsions and coma [8]. Oxidative stress plays a major role in brain ageing [32], ischemia [33], stroke [34], Alzheimer's [35] and schizophrenia by mitochondrial dysfunction [36]. Based on previous studies, phosphine generated from ALP is a mitochondrial toxin that inhibits cellular respiration and induces oxidative stress, resulting in free radical generation. Phosphine gas produced by ALP causes oxidative stress and free radicals in cell mitochondria by blocking cellular respiration and causing cellular respiration inhibition. ALP toxicity has been evaluated in multiple experimental studies, and oxidative stress has emerged as one of its most crucial mechanisms [37],[38]. We observed biochemical, molecular and histological changes in brain tissue after subacute ALP exposure caused oxidative stress as an essential effect in poisoned rats.

The nervous system is at a high risk of free radical-induced injury because it is rich in polyunsaturated fatty acid side chains, and it has high oxygen tension and relatively poor antioxidant capacity [39]. Free radicals increase the peroxidation rate of polyunsaturated fatty acids, resulting in membrane damage. ALP may damage neuronal tissue by disrupting membrane structure and function, leading to immediate effects and subsequent afflictions [8]. Lipid peroxidation is when oxidants such as free radicals attack lipids containing carbon-carbon double bonds. Under lipid peroxidation, the extent of oxidative damage suppresses repair capacity and induces necrosis-programmed cell death or apoptosis [40]. MDA is the most mutagenic product in the lipid peroxidation field among the many different aldehydes that form during lipid peroxidation [41]. Our study showed that ALP increased the brain MDA level, suggesting phosphine's production of free radicals.

Also, CUR and nanomicelle-CUR treatments reduced the MDA level in ALP-induced brain toxicity. MDA levels of mice hearts were increased by ALP, according to Gouda et al. Additionally, Moringa oleifera (Lam) extracts also reduced the heart MDA level in the presence of ALP, suggesting the extracts could be used to reduce heart toxicity from ALP [6]. In another study by Afolabi et al., ALP increased MDA levels in the ovarian tissues of rats. In contrast, the level of MDA was decreased in the group treated with antioxidant Hesperidin and ALP. Hesperidin may enhance antioxidant defenses when ALP is present [42]. The study of Tehrani et al. also showed that the MDA level was higher in patients with ALP poisoning. At the same time, it was lower in those treated with ALP and antioxidant N-acetylcysteine, suggesting N-acetylcysteine may reduce the toxic effects of ALP [43].

The TTG of proteins as a sensitive indicator plays a significant role in response to antioxidant response [44]. In the present study, the lowest amount of TTG belonged to the rats receiving free ALP. In addition, the level of TTG was increased when rats received CUR and especially nanomicelle-CUR in the presence of ALP. CUR and, in particular, nanomicelle-CUR can be used to reduce the toxicity of ALP within the brain by increasing TTG. An animal study conducted by Fakhraei et al. showed that rats with ALP poisoning had a lower TTG level in heart tissue, whereas rats treated with nanomicelle-CUR or CUR had higher TTG levels [10]. We reported previously that the liver TTG level was reduced in the group of rats receiving ALP. In contrast, it was increased after the rats were treated with ALP in the presence of CUR or nanomicelle-CUR [1].

The TAC, as a valuable indicator of the body's antioxidant status, has been related to the development of various diseases [45]. Comparing the ALP-treated rats to the control group, this study showed that the TAC level was lower in the ALP-treated rats. Moreover, rats given CUR and nanomicelle-CUR in the presence of ALP had a higher TAC level. As reported by Kariman et al., the plasma TAC level decreased when ALP was administered to patients [2]. According to Afolabi et al., the TAC level in rat testicular tissue was reduced following treatment of the testis with ALP, whereas the level was increased after treatment with antioxidant hesperidin [46]. Also, it has been found that the TAC level decreased in patients with ALP poisoning while N-acetylcysteine antioxidants increased it [43].

The ROS family includes H2O2, a chemically reactive molecular species derived from oxygen. Oxidative stress is controlled in part by intrinsic antioxidant enzymes. SOD is one of the primary cellular antioxidants which can catalyze the conversion of superoxide anion to H2O2 [47]. As a result of our study, brain SOD levels decreased in the ALP group, whereas it increased in ALP groups treated with CUR or nanomicelle-CUR. Compared with ALP, nanomicelle-CUR treatments in the presence of ALP increased the level of SOD in rat liver mitochondria, according to our previous study [1]. An increase in heart SOD was reported by Fakhraei et al. using ALP, while nanomicelle-CUR was successful in increasing heart SOD level [10]. Rajeswari's study found that CUR increased the specific activity of SOD, CAT and lipid peroxidation in mice's striatum and midbrain. According to their findings, CUR could potentially be neuroprotective against oxidative stress [48]. Irinotecan (CPT-11), an important anticancer drug, decreased the level of SOD and caused oxidative damage in the heart tissue of rats. Still, CUR treatment reversed these toxic effects by raising the SOD level [47].

SIRT1 is an enzyme involved in the deacetylation of nicotinamide adenine dinucleotide (NAD+); it belongs to the mammalian sirtuin family, which is widely studied. SIRT1 is nearly exclusively found in the nucleus, where it plays a significant role in deacetylating histones and inhibiting the transcription [49]. In addition to regulating many biological processes, sirtuins modify the structural organization of the brain through axon extension, dendritic plasticity and embryonic neurogenesis [50],[51].

Reactive stress appears closely related to SIRT1 in the brain tissue [52]. The role of SIRT1 in protection from oxidative stress has received considerable attention. An important mechanism involved in this process is SIRT1/FOXO [53],[54].

The FOXOs belong to the Forkhead family of transcriptional factors, among which FoxO1 and FoxO3 are the most common [55],[56]. Many cellular functions, such as inhibiting oxidative stress, are activated when FOXOs move into the nucleus and start protein expression. This oxidative stress resistance mechanism involves the connection between SIRT1 and FOXO [57],[58]. Cell viability against oxidative stress depends on FOXO1, FOXO3a and SIRT-1. Through stimulation of oxidative stress, SIRT-1 and FOXO3 have been shown to form a complex, and SIRT-1 induces resistance to oxidative stress by deacetylating FOXO3. Sirt1 acts by deacetylating FOXO1 and reducing the oxidative stress response [59]. SIRT1 and FOXO1 gene expression are regulated by the positive feedback mechanism [60].

Results revealed that expression of SIRT1 mRNA in the damaged brain tissue was significantly reduced compared to healthy brain tissues, and deacetylated FOXO3 level was also considerably decreased in injured brain tissue compared to the healthy control [61]. Moreover, it has been seen that the FOXO1 expression and activity were downregulated in oxidative stress-induced injuries in the brain tissue [62].

The present study showed a significant decrease in gene expression of SIRT1, FOXO1 and FOXO3 following subacute exposure to ALP in rats' brain tissues. Additionally, ALP-exposed brain tissues showed a significant decrease in CAT and GPX levels, which was observed parallel to the decline and deacetylation of FOXO3 protein after sub-acute exposure to this compound. In the presence of curcumin and nanocurcumin, SIRT1 gene expression was elevated, resulting in elevated levels of deacetylated FOXO3 protein and CAT and GPx production. After subacute exposure to ALP, the damaged indicators were reversed, suggesting that the SIRT1/FOXO3 signaling pathway is essential for improving the antioxidant capacity of brain tissue and reducing oxidative stress. In many studies, curcumin has decreased oxidative stress and activated SIRT1. Curcumin may prevent or treat oxidative stress-related injuries by increasing SIRT1 levels [63]–[66]. The upregulation of FOXO1 mRNA by curcumin has also been demonstrated [67],[68].

Histopathologically, we have found that ALP may lead to oxidative and histological effects in the brain and reduced cell density showed that nanomicelle-CUR exhibits brain-protective properties associated with low hippocampal CA1 pyramidal cells or high cell density. According to Fallah et al., the number of pyramidal cells in hippocampal CA1 was decreased in rats exposed to arsenic, and CUR and N-acetylcysteine treatments improved these abnormalities [69]. In addition, a study by Ciftci et al. reported that CPT-11 treated rats had oxidative and histological damage in the cardiac tissue, such as inflammatory cell infiltration, hemorrhage, vascular congestion and vacuolization. At the same time, CUR prevented this damage [47].

## 5. Conclusion

In conclusion, this study established that ALP brought about significant oxidative damage in the brain. Additionally, CUR administered in nanomicelle form protected against ALP-induced brain damage.
